# Thermodynamic Analysis of an Irreversible Maisotsenko Reciprocating Brayton Cycle

**DOI:** 10.3390/e20030167

**Published:** 2018-03-05

**Authors:** Fuli Zhu, Lingen Chen, Wenhua Wang

**Affiliations:** 1 Institute of Thermal Science and Power Engineering, Naval University of Engineering, Wuhan 430033, China; 2 Military Key Laboratory for Naval Ship Power Engineering, Naval University of Engineering, Wuhan 430033, China; 3 College of Power Engineering, Naval University of Engineering, Wuhan 430033, China

**Keywords:** finite-time thermodynamics, irreversible Maisotsenko reciprocating Brayton cycle, power output, efficiency

## Abstract

An irreversible Maisotsenko reciprocating Brayton cycle (MRBC) model is established using the finite time thermodynamic (FTT) theory and taking the heat transfer loss (HTL), piston friction loss (PFL), and internal irreversible losses (IILs) into consideration in this paper. A calculation flowchart of the power output (P) and efficiency (η) of the cycle is provided, and the effects of the mass flow rate (MFR) of the injection of water to the cycle and some other design parameters on the performance of cycle are analyzed by detailed numerical examples. Furthermore, the superiority of irreversible MRBC is verified as the cycle and is compared with the traditional irreversible reciprocating Brayton cycle (RBC). The results can provide certain theoretical guiding significance for the optimal design of practical Maisotsenko reciprocating gas turbine plants.

## 1. Introduction

The revolutionary Maisotsenko cycle (M-cycle), utilizing the psychrometric renewable energy from the latent heat of water evaporating, was firstly provided by Maisotsenko in 1976. The configuration (the key component, Maisotsenko air saturator particularly) and the working process of the M-cycle were introduced in Reference [1]. The broad prospective applications of the M-cycle in heating ventilation, air-conditioning, the power industry, water distillation, and heat recovery have been illustrated in detail in References [2,3,4,5]. The status of the application of M-cycle expansion to the gas turbine cycle (Brayton cycle) was shown, and a comparative analysis with the traditional gas turbine cycle was carried out in References [3,4,5]. The result verified that the Maisotsenko gas turbine cycle (MGTC) is superior to the humid air turbine cycle and Brayton cycle in P and η [6]. A model of MGTC with a polytropic process was established by Saghafifar and Gadalla [7] and the influences of various operating parameters on the overall performance of the cycle were studied by taking P and η as the optimization objectives. Furthermore, based on the MGTC, the P and η of the Maisotsenko open gas turbine bottoming cycle were reported in Reference [8]. Khalatov et al. [9] analyzed the performance of the sub-atmospheric reverse Brayton cycle with waste heat regeneration according to the M-cycle, and the result demonstrated that a higher η could be attained. Saghafifar and Gadalla [10] recently investigated the optimal thermo-economic performance of the hybrid solar Maisotsenko bottoming cycle. Nevertheless, all of the research works mentioned above have been performed based on classical thermodynamics.

Since FTT [11,12,13,14,15,16,17,18,19,20,21,22,23,24] has been applied to the performance analyses and optimizations for gas turbine cycles, plentiful achievements in scientific research have been obtained. Amass of work about the performance analyses and optimizations using FTT for simple [25,26,27,28,29], regenerated [30,31,32,33,34], intercooled and regenerated [35,36], combined Brayton and inverse Brayton [37], multi-stage intercooled and regenerated [38] and reciprocating Brayton cycles [39,40], by selecting the P, η, and ecological function as optimization objectives, considering HTL and/or IILs has been published. Based on References [7,40], an irreversible model of the Maisotsenko reciprocating Brayton cycle (MRBC) will be established using the FTT theory with considerations of HTL, piston friction loss (PFL), and IILs in this paper. A calculation flowchart of the P and η of the cycle will be provided, and the effects of the pressure ratio, the maximum temperature of the cycle, the outlet temperature of humid air at the air saturator (AS), the outlet temperature of exhaust gas at AS, and the MFR of the injection of water to the performance of cycle will be analyzed by detailed numerical examples.

## 2. Cycle Model and Performance Analyses of Irreversible Maisotsenko Reciprocating Brayton Cycle

**Figure 1:**
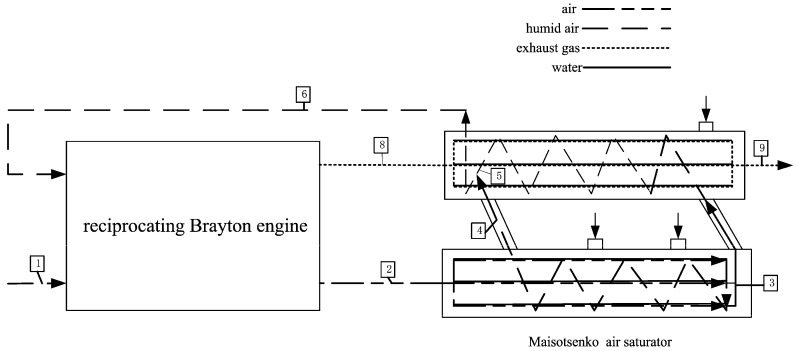
Schematic diagram of the irreversible Maisotsenko reciprocating Brayton cycle.

**Figure 2:**
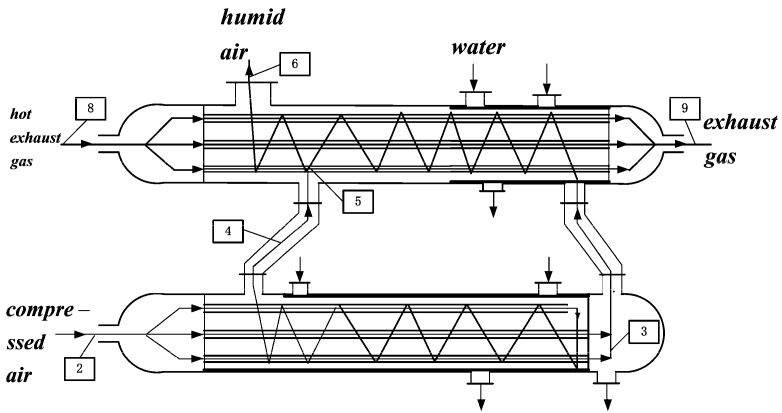
M-Cycle shell and tube air saturator [5].

The configuration of irreversible MRBC is shown in Figure 1 [7]. The operational scheme of AS is shown in Figure 2 [5]. Firstly, fresh air is compressed adiabatically, when the air, after being compressed, is separated into three strands and enters the bottom section of AS for heating and humidifying. All of the air streams are chilled down to the dew point temperature of the inlet air of AS by evaporation of water indirectly. One part of the airstreams is heated up and humidified to the saturation point sequentially in the bottom section of AS. The others blend and arrive at the above section of AS directly. Cooled dry air is heated up and humidified by utilizing the available waste heat from the expander exhaust gas afterwards in the top section of AS. In succession, humid air strands are mixed together and heated up before entering the cylinder in the above section of AS.

**Figure 3:**
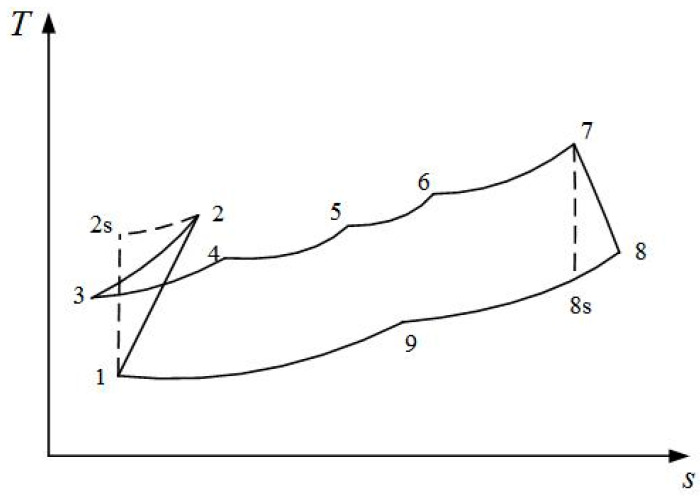
T-s diagrams for the irreversible Maisotsenko reciprocating Brayton cycle.

The T-s diagram for irreversible MRBC is represented in Figure 3. The compression process 1→2s is adiabatic, while 1→2 takes the all irreversible losses including PFL into consideration, the heat rejection process 2→3 in the bottom section of AS is constant pressure, the heat addition and humidification process 3→4 of one part of the airstreams in the bottom section of AS is constant pressure, the heat addition and humidification process 4→5 of the others strands in the top section of AS is constant pressure, the heat addition and humidification process 5→6 of all of the humid airstreams in the top section of AS is constant pressure, the heat addition process 6→7 of humid air is constant pressure, the expansion process 7→8s is adiabatic while 7→8 takes all the irreversible losses including PFL into consideration, the heat rejection process 8→9 of expander exhaust gas in the top section of AS is constant pressure, and the heat rejection process 9→1 of expander exhaust gas is constant pressure.

According to the property of saturated humid air, the saturation pressure can be decided as:

(1) $$p_{s}=\alpha_{s}p$$

where p is the pressure of humid air, and $\alpha_{s}$ is the mole fraction of steam in humid air which can be determined as:

(2) $$\alpha_{s}=n_{s}/(n_{s}+n_{a})$$

where $n_{s}$ and $n_{a}$ are the mole numbers of steam and dry air, respectively, and they can be determined as:

(3) $$n_{s}\;=\;m_{s}/M_{s}$$

(4) $$n_{a}\;=\;m_{a}/M_{a}$$

where $m_{s}$ and $m_{a}$ are the MFR of steam and dry air, respectively, and $M_{s}$ and $M_{a}$ are the molecular weights of steam and dry air, respectively.

The MFR of saturated or superheated steam is [41]:

(5) $$m_{s}=0.622p_{s}/(p-p_{s})$$

The enthalpy of steam can be determined by [42]:

(6) $$h_{s}=f(p_{s},T,x)$$

where x(0≤x≤1) is the dryness of steam. When x=1, the steam is saturated or superheated, while when 0<x<1, the steam is in the gas-liquid phase. Here, x can be calculated by [41]:

(7) $$x=(s-s_{\mathrm{wa}})/(s_{s}-s_{\mathrm{wa}})$$

where $s_{w}$ and $s_{s}$ are the entropies of the saturated water and saturated steam, respectively.

The enthalpy of dry air can be determined by [41]:

(8) $$h_{a}=m_{a}C_{P}(T-273.15)$$

Being the maximum temperature $T_{7}$, the initial state of MFR of steam $m_{1s}$, the temperature $T_{1}$, and the pressure $p_{1}$ are given, the MFRs at different states can be written as:

(9) $$m_{1s}=m_{2s}$$

(10) $$m_{3s}=2m_{2s}/3$$

(11) $$m_{4s}=(m_{2s}+3m_{\mathrm{winB}})/3$$

(12) $$m_{6s}=m_{7s}=m_{8s}=m_{9s}=m_{4s}+m_{3s}+m_{\mathrm{winA}}$$

where $m_{\mathrm{winB}}$ and $m_{\mathrm{winA}}$ are the MFRs of the injection of water in the bottom section and upper part of AS, respectively. The MFR of dry air is constant.

The initial state of pressure of steam $p_{1s}$ can be determined by Equation (5)

(13) $$p_{1s}=\beta p_{1}(1+\beta )$$

where $\beta =m_{1s}/0.622$.

If we can make the assumption that there is no pressure loss, the pressure ratio of compressor is:

(14) $$\pi =p_{2s}/p_{1}=p_{7}/p_{8s}$$

y is defined as the compressor isentropic temperature ratio, and:

(15) $$y=T_{2s}/T_{1}=T_{7}/T_{8s}={\left(p_{2s}/p_{1}\right)}^{m}={\left(p_{7}/p_{8s}\right)}^{m}=\pi^{m}$$

where m=(k−1)/k, and k is the air adiabatic exponent in the compressor.

It can be determined from Equation (15) that:

(16) $$T_{2s}=yT_{1}$$

(17) $$T_{8s}=T_{7}/y$$

The efficiencies of the irreversible compression and irreversible expansion processes can be defined as:

(18) $$\eta_{c}=\left(T_{2s}-T_{1}\right)/\left(T_{2}-T_{1}\right)$$

(19) $$\eta_{e}=\left(T_{7}-T_{8}\right)/\left(T_{7}-T_{8s}\right)$$

All the irreversible losses including PFL are associated with two efficiencies.

It is assumed that steam does not undergo phase change after expansion, according to the ideal gas state equation:

(20) $$pV=R_{g}T$$

Thus, $p_{2}$ and $p_{8}$ can be determined, respectively, as:

(21) $$p_{2}=T_{2}p_{2s}/T_{2s}$$

(22) $$p_{8}=T_{8}p_{8s}/T_{8s}$$

The pressures of humid air and the steam at different conditions can be written as:

(23) $$p_{3}=p_{4}=p_{5}=p_{6}=p_{7}=p_{2}$$

(24) $$p_{9}=p_{8}$$

(25) $$p_{2s}=p_{3s}=\pi p_{1s}T_{2}/T_{2s}$$

(26) $$p_{6s}=p_{7s}$$

(27) $$p_{9s}=p_{8s}=p_{7s}T_{2}/\pi T_{2s}$$

The primary mission of the bottom section of AS is to obtain the cooled air, and therefore the air which is saturated for sure at the outlet of the bottom section of AS with an excess injection of water. Consequently, from Equation (1), we can obtain:

(28) $$p_{4s}=\alpha_{4}p_{4}$$

On the basis of the feature of AS, the compressed air can be chilled down to the dew temperature under the pressure $p_{3s}$.Owing to the steam being saturated at state 4, $T_{4}$ can be calculated by $p_{4s}$.

In turn, parameters $h_{2s}$, $h_{3s}$, and $h_{4s}$ can be determined by Equations (6) and (7), and parameters $h_{2a}$, $h_{3a}$, and $h_{4a}$ can be calculated by Equation (8) successively.

Consequently, energy balance for the bottom section of AS can be represented by:

(29) $$m_{2s}h_{2s}+m_{2a}h_{a2}+m_{\mathrm{winB}}h_{\mathrm{wa}}=m_{3s}h_{3s}+m_{3a}h_{3a}+m_{4s}h_{4s}+m_{4a}h_{4a}$$

where $h_{\mathrm{wa}}$ is the enthalpy of the injected water, $m_{\mathrm{winB}}$ can be estimated by an iterative procedure, $p_{4s}$ can be obtained according to Equation (28), and $T_{4}$ can be determined by $p_{4s}$.

The MFR of injected water is the maximum when the steam is saturated at state 6, according to Equation (1).

(30) $$p_{6s}=\alpha_{6}p_{6}$$

In accordance with the characteristics of AS, the temperature of expander exhaust gas can be chilled down to the dew temperature of the inlet air of AS:

(31) $$T_{9}=T_{2\mathrm{dew}}$$

Successively, $h_{8s}$ and $h_{9s}$ can be estimated by Equations (6) and (7). $h_{8a}$ and $h_{9a}$ can be determined by Equation (8). If $T_{6}>T_{8}$, the AS cannot chill down the exhaust gas, and for solving the problem, it is assumed that:

(32) $$T_{6}=T_{8}-15$$

An energy balance for the top section of AS can be written as:

(33) $$\begin{array}{l} m_{3s}h_{3s}+m_{3a}h_{3a}+m_{4s}h_{4s}+m_{4a}h_{4a}+m_{\mathrm{winA}}h_{\mathrm{wa}}+m_{8s}h_{8s}+m_{8a}h_{8a}\\ \;\;\;\;=m_{9s}h_{9s}+m_{9a}h_{9a}+m_{6s}h_{6s}+m_{6a}h_{6a} \end{array}$$

According to the same principle of the bottom section of AS, $T_{6}$ can be calculated by an iterative procedure.

The heat addition rate and heat rejection rate of cycle are given as follows, respectively:

(34) $$Q_{\mathrm{in}}=Q_{67}=m_{7s}h_{7s}-m_{6s}h_{6s}+m_{a}h_{7a}-m_{a}h_{6a}$$

(35) $$Q_{\mathrm{out}}=Q_{91}=m_{9s}h_{9s}-m_{1s}h_{1s}+m_{a}h_{9a}-m_{a}h_{1a}$$

For an ideal MRBC model, HTL is not considered. Nevertheless, for a real MRBC, the HTL between the high temperature of humid air and the environment must be taken into consideration. On the basis of references [43,44], the HTL is:

(36) $$Q_{\mathrm{in}}=A_{1}-B_{1}[(T_{6}+T_{7})/2-T_{0}]$$

where the rate of heat release by combustion is $A_{1}$, and the coefficient of heat leakage of the cylinder wall is $B_{1}$.

According to Equation (35), the rate of heat leakage is:

(37) $$Q_{\mathrm{leak}}=\frac{B}{2}(T_{6}+T_{7}-2T_{0})$$

where $B=B_{1}/2$ is a constant and $T_{0}$ is the temperature of the environment.

For the practical cycle, there is PFL caused by the piston motion. The PFL of processes 1-2 and 7-8 have been included as $\eta_{c}$ and $\eta_{e}$. According to References [45,46], one can assume that μ is the friction coefficient of exhaust stroke friction, 3μ is the friction coefficient of intake stroke, and if the function between friction force and velocity is linear [47]:

(38) $$f_{\mu }=-\mu v=-\mu dX/dt$$

where X is the displacement of the piston, and v is the piston velocity. Therefore, the power loss is:

(39) $$P_{\mu }=\frac{dW_{\mu }}{dt}=4\mu \frac{dX}{dt}\frac{dX}{dt}=4\mu v^{2}$$

where $W_{\mu }$ is the lost work caused by friction.

For the four-stroke engines, the distance of the piston travels per cycle is:

(40) $$4L=4(X_{1}-X_{2})$$

where $X_{1}$ and $X_{2}$ are the maximum and minimum piston positions, respectively.

Consequently, the average velocity of the piston is:

(41) $$\bar{v}=4LN$$

where N is the cycle numbers per second.

Thus, the lost power caused by friction can be written as:

(42) $$P_{\mu }=4\mu {\left(4LN\right)}^{2}=64\mu {\left(LN\right)}^{2}$$

Consequently, the P and η of the cycle, respectively, are:

(43) $$P=Q_{\mathrm{in}}-Q_{\mathrm{out}}-P_{\mu }$$

(44) $$\eta =P/(Q_{\mathrm{in}}+Q_{\mathrm{leak}})$$

## 3. Numerical Examples and Discussion

On the basis of Reference [7], the relevant parameters are selected as $C_{P}=1.005\;\mathrm{kJ}/(\mathrm{kg}\cdot K)$, $T_{1}=288\;K$, $T_{0}=288\;K$, $m_{a}=1\;\mathrm{kg}/s$, k=1.4, B=0.5 kW/K, $T_{\mathrm{water}}=298\;K$, N=30, μ=0.9kg/s, and X=0.06m. By using detailed numerical calculations, the relations of P−π, η−π, P−η are obtained. The effects of the maximum temperature $T_{7}$, outlet temperature $T_{6}$ of humid air in the top section of AS, outlet temperature $T_{9}$ of exhaust gas in the top section of AS, MFR $m_{\mathrm{win}}$ of the injection of water to the cycle, and some other design parameters on cycle performances are analyzed in this section.

Taking the feature of AS into consideration, if $T_{4}<T_{3}$, air cannot be heated and humidified to a saturation state in the bottom section of AS, which is against the design principle of AS. If $T_{6}<T_{4}$, the saturated steam which comes from the bottom section of AS cannot continue to be heated up and humidified, which is against the assumption. If $T_{8}<T_{6}$, AS does not have the capacity of regeneration. If the MFR of the injection of water to the cycle is more than the MFR of the saturation state, the humid air at the outlet of AS is unsaturated, which is against the assumption above. 

**Figure 4:**
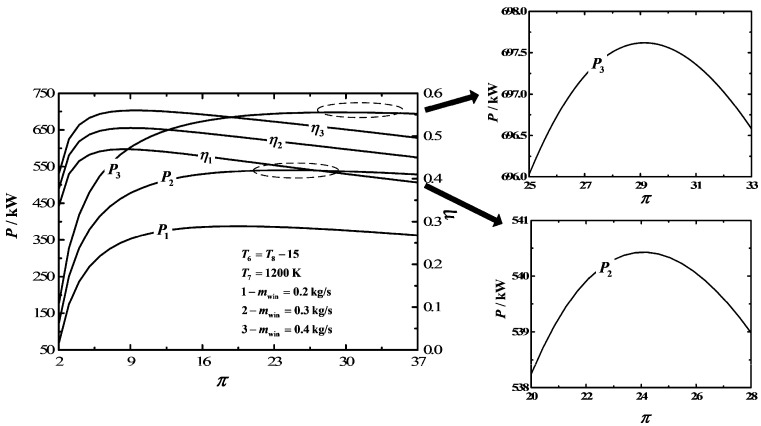
Influences of $m_{\mathrm{win}}$ on the characteristics of P−π and η−π.

Figure 4 depicts the influences of MFR ($m_{\mathrm{win}}$) of the injection of water to the cycle on P−π and η−π characteristics. The results show that if the range of π is 2-36, the characteristic curve of P−π is a parabolic-like one, which has one maximum P point ($P_{\max}$). Moreover, the optimal pressure ratio ($\pi_{P,\max}$) corresponding to $P_{\max}$ and $P_{\max}$ increases as $m_{\mathrm{win}}$ increases. The characteristic curve of η−π is also a parabolic-like one, which has one maximum η point ($\eta_{\max}$). Furthermore, the optimal pressure ratio ($\pi_{\eta ,\max}$) corresponding to $\eta_{\max}$ and $\eta_{\max}$ increases as $m_{\mathrm{win}}$ increases.

**Figure 5:**
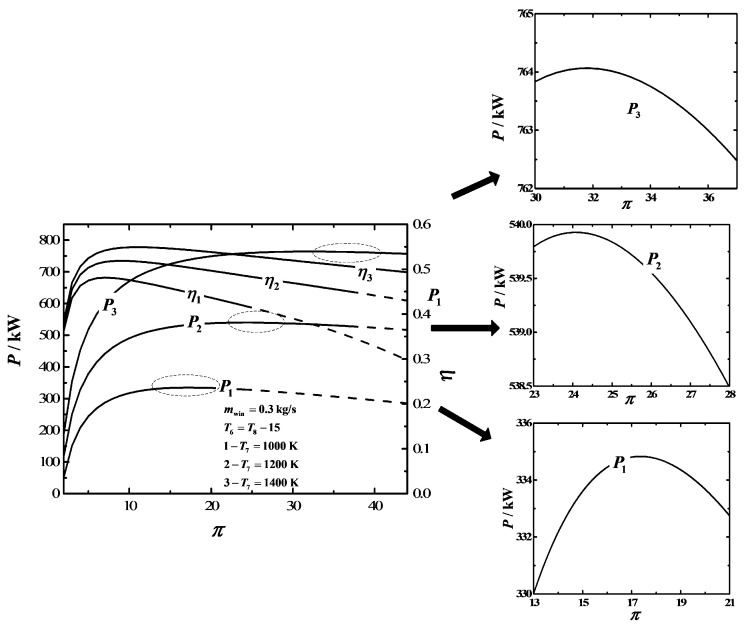
Influences of $T_{7}$ on the characteristics of P−π and η−π.

Figure 5 depicts the influences of the maximum temperature ($T_{7}$) on P−π and η−π characteristics. The results show that the range of π is 2-23 if $T_{7}=1000\;K$, the range of π is 2-36 if $T_{7}=1200\;K$, and the range of π is 2-43 if $T_{7}=1400\;K$. The characteristic curve of P−π is a parabolic-like one, which has one maximum P point. Moreover, $\pi_{P,\max}$ and $P_{\max}$ increase as $T_{7}$ increases. The characteristic curve of η−π is also a parabolic-like one, which has one maximum η point. Furthermore, $\pi_{\eta ,\max}$ and $\eta_{\max}$ increase as $T_{7}$ increases.

**Figure 6:**
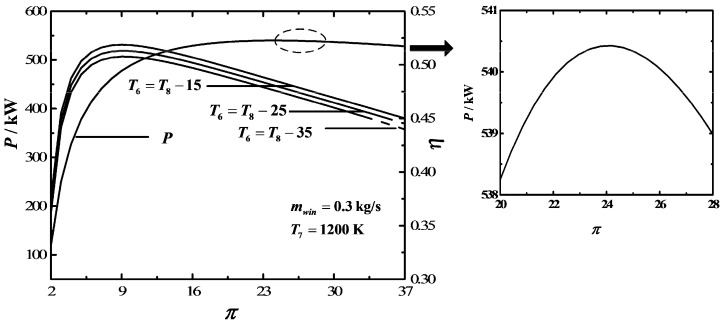
Influences of $T_{6}$ on the characteristics of P−π and η−π.

Figure 6 depicts the influences of the outlet temperature ($T_{6}$) of the bottom section of AS on P−π and η−π characteristics. The results show that the range of π is 2-32 if $T_{6}=T_{8}-35$ and the range of π is 2–36 if $T_{6}=T_{8}-25$. Within this range, $T_{6}$ makes no difference to P. However, the characteristic curve of P−π is a parabolic-like one, which has one maximum P point. The characteristic curve of η−π is also a parabolic-like one, which has one maximum η point. Furthermore, $\pi_{\eta ,\max}$ and $\eta_{\max}$ increase as $T_{6}$ increases.

**Figure 7:**
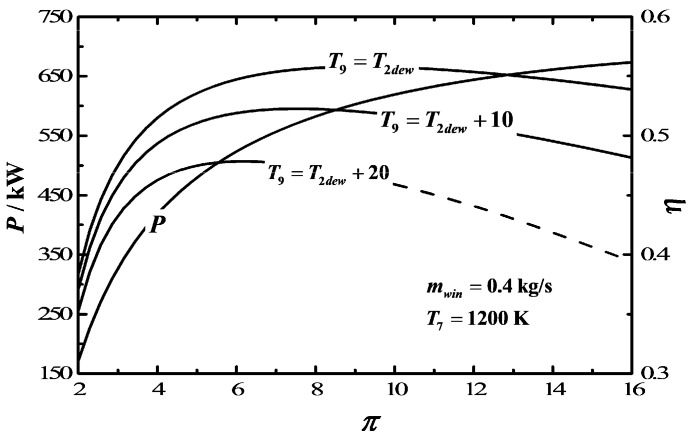
Influences of $T_{9}$ on the characteristics of P−π and η−π.

Figure 7 depicts the influences of the outlet exhaust gas temperature ($T_{9}$) of AS on P−π and η−π characteristics. The results show that the range of π is 2-10 if $T_{9}=T_{2dew}+20$ and the range of π is 2–16 if $T_{9}=T_{2dew}+10$. Within this range, $T_{9}$ makes no difference to P, and P increases when π increases. The characteristic curve of η−π is a parabolic-like one, which has one maximum η point. Moreover, $\pi_{\eta ,\max}$ and $\eta_{\max}$ increase as $T_{9}$ decreases.

## 4. Comparison with the Traditional Irreversible Reciprocating Brayton Cycle

**Figure 8:**
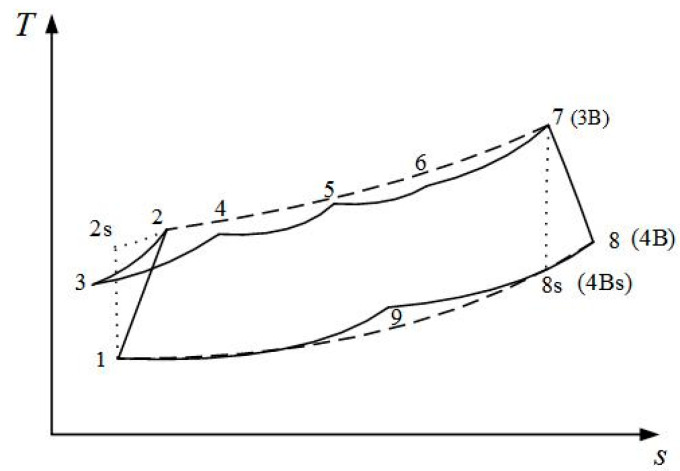
T-s diagram for the irreversible Maisotsenko reciprocating Brayton cycle and reciprocating Brayton cycle.

The T-s diagram for the traditional irreversible RBC is shown in Figure 8. The solid line represents the irreversible MRBC, and the broken line represents the traditional irreversible RBC. The heat addition process 2→3B is a constant pressure process, the expansion process 3B→4Bs is an adiabatic process while 3B→4B takes the all irreversible losses including PFL into consideration, and process 4B→1 is a constant pressure exothermic process.

Similar to the irreversible MRBC, the pressure ratio of compressor π is:

(45) $$\pi =p_{2}/p_{1}=p_{3B}/p_{4\mathrm{Bs}}$$

y is defined as the compressor isentropic temperature ratio, and:

(46) $$y=T_{2}/T_{1}=T_{3B}/T_{4\mathrm{Bs}}={\left(p_{2}/p_{1}\right)}^{m}={\left(p_{3B}/p_{4\mathrm{Bs}}\right)}^{m}=\pi^{m}$$

where m=(k−1)/k, k is the air adiabatic exponent.

The primary temperature $T_{1}$ and the maximum temperature $T_{3B}$ are equal to the irreversible MRBC:

(47) $$T_{3B}=T_{7}$$

The efficiency of the irreversible expansion process $\eta_{e}$ for the process 3B→4B is:

(48) $$\eta_{e}=\left(T_{3B}-T_{4B}\right)/\left(T_{3B}-T_{4\mathrm{Bs}}\right)$$

For the HTL and PFL of traditional irreversible RBC, the model of irreversible MRBC still holds. Therefore, Equations (35)–(41) can be applied to the traditional irreversible RBC.

Consequently, the P and η of the traditional cycle, respectively, are:

(49) $$P=Q_{\mathrm{in},B}-Q_{\mathrm{out},B}-P_{\mu }=m_{a}C_{P}[T_{3B}(2-\eta_{c})/\eta_{c}-T_{1}]-64\mu {\left(Ln\right)}^{2}$$

(50) $$\eta =P/(Q_{\mathrm{in}}+Q_{\mathrm{leak}})$$

**Figure 9:**
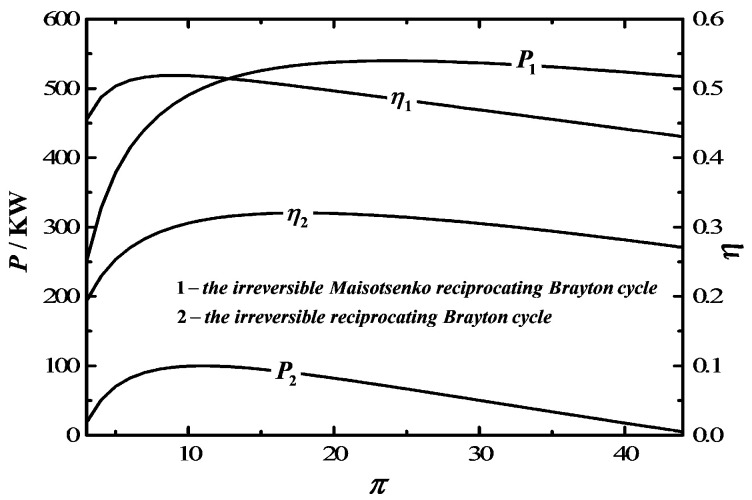
Comparison of P−π and η−π between the Maisotsenko reciprocating Brayton cycle and reciprocating Brayton cycle.

Figure 9 shows the comparison between the irreversible MRBC and traditional irreversible RBC in P and η, and the results show that the irreversible MRBC is superior to the traditional irreversible RBC in both P and η.

## 5. Conclusions

Based on References [7,40], a model of irreversible MRBC is established using the FTT theory in this paper. A calculation flowchart of P and η of the cycle is given, and the effects of the maximum temperature, the outlet temperature of humid air in the top section of AS, the outlet temperature of exhaust gas in the top section of AS, and the MFR of the injection of water to the cycle, are analyzed by using detailed numerical examples. The results are indicative that the maximum temperature and the MFR of the injected water have great influences on P and η. Moreover, the outlet temperature of the bottom section of AS and the outlet exhaust gas temperature of AS have less influence on the power output, but greatly affect η. It is also demonstrated that the irreversible MRBC is superior to the traditional irreversible RBC in terms of P and η. The results can afford guidance for the practical optimization of Maisotsenko reciprocating gas turbine plants.

## Nomenclature

$A_{1}$	heat released rate by combustion
$B_{1}$	heat leakage coefficient of combustion charmer
$C_{P}$	specific heat at constant pressure
$f_{\mu }$	friction force
h	enthalpy
k	air adiabatic exponent
L	the distance of the piston travels per cycle
M	molecular weight
m	mass flow rate
N	cycle index per second
n	mole number
P	power output
p	pressure
Q	heat rate of addition or rejection
R_g_	ideal gas constant
s	entropy generation
T	temperature
t	time
V	volume
v	piston speed
W	work
X	displacement of piston
x	dryness of steam
y	compressor isentropic temperature rate

Greek letters

α	mole fraction of steam in humid air
$\eta_{c}$	efficiencies of irreversible compression
$\eta_{e}$	efficiency of irreversible expansion
μ	friction coefficient
π	compression ratio

Subscripts

a	air
B	reciprocating Brayton cycle
dew	dew point temperature
in	heat addition
leak	leakage
max	maximum
out	heat rejection
P, max	maximum power out
s	steam
wa	water
win	air saturator water inlet
winA	above air saturator water inlet
winB	below air saturator water inlet
η, max	maximum efficiency
μ	effect of friction
1, 2, 3, 4, 5, 6, 7, 8, 9	cycle state points

## Abbreviations

The following abbreviations are used in this manuscript:

AS	air saturator
FTT	finite time thermodynamic
HTL	heat transfer loss
IIL	internal irreversible loss
MFR	mass flow rate
MGTC	Maisotsenko gas turbine cycle
MRBC	Maisotsenko reciprocating Brayton cycle
PLF	piston friction loss
RBC	reciprocating Brayton cycle
